# Investigation of YSO–SiPM detector response for alpha spectroscopy applications

**DOI:** 10.12688/openreseurope.24095.1

**Published:** 2026-09-18

**Authors:** Gadir Ahmadov, Sebuhi Nuruyev, Daniyar Berikov, Arzu Mammadli, Azer Sadygov, Alisher Temirzhanov, Kairat Mendibayev, Murad Kenessarin, Ramil Akbarov, Farid Ahmadov

**Affiliations:** 1 Nuclear Research Department, Innovation and Digital Development Agency, Abşeron, AZ1069, Azerbaijan; 2 Azerbaijan University of Architecture and Construction, Baku, AZ1073, Azerbaijan; 3 Innovative Electronics and Detectors LLC, Baku, AZ1021, Azerbaijan; 4 Institute of Physics - Ministry of Science and Education, Baku, AZ1073, Azerbaijan; 5 Khazar University, Baku, AZ1096, Azerbaijan; 6 The Institute of Nuclear Physics, Almaty, 050032, Kazakhstan

**Keywords:** alpha spectroscopy, silicon photomultiplier, MPPC, YSO scintillator, radiation detector

## Abstract

**Background:**

Alpha-particle spectroscopy is widely used in nuclear physics, radiation monitoring, safeguards, and environmental measurements. Conventional silicon detectors provide excellent spectrometric performance; however, their application in portable or harsh-environment instrumentation may be limited by mechanical fragility, radiation sensitivity, and operational constraints. Scintillator-based detectors coupled to silicon photomultipliers (SiPMs) represent a promising alternative because of their compactness, robustness, and simplified integration with modern electronics. Despite the favorable scintillation properties of yttrium orthosilicate (YSO), its applicability for alpha-particle spectroscopy has received limited investigation.

**Methods:**

The response of a YSO scintillator coupled to a Hamamatsu S13360–3050 multi-pixel photon counter (MPPC) array was investigated using alpha particles emitted from a ^238^Pu source. A 10 × 10 × 10 mm ^3^ YSO crystal was coupled to the SiPM array and read out using CAEN digitization electronics. Alpha-particle energies in the approximate range from 0.4 MeV to 5.5 MeV were obtained by varying the air-layer thickness between the radioactive source and detector. Signal acquisition was performed using a CAEN DT5720 digitizer operated in self-trigger mode, while offline waveform processing was performed using ROOT-based software.

**Results:**

The YSO–SiPM detector exhibited stable and approximately linear response characteristics across the investigated energy interval. The measured deviation from linearity was approximately 3%. The experimentally determined alpha-particle flight distance in air was measured as 40.58 ± 0.87 mm, in good agreement with SRIM simulations predicting approximately 42 mm. The measured energy resolution varied from approximately 10.5% at 5.5 MeV to approximately 21% at lower alpha-particle energies near 0.4 MeV. Alpha-particle signals remained clearly distinguishable down to approximately 500 keV.

**Conclusions:**

The obtained results demonstrate that the YSO-SiPM configuration represents a compact and mechanically robust detector solution for alpha-particle spectroscopy. Although the energy resolution remains inferior to conventional silicon alpha detectors, the investigated system offers important practical advantages including operation under ambient conditions, simplified electronics integration, scalability, and suitability for portable or harsh-environment nuclear instrumentation.

## Plain Language summary

Alpha particles are emitted by radioactive materials and are commonly measured in nuclear physics, environmental monitoring, radiation safety, and nuclear-security applications. Traditional alpha-particle detectors based on silicon technology provide very accurate energy measurements, but they can be fragile, expensive for large-area systems, and difficult to use in portable or harsh-environment instruments.

In this study, we investigated an alternative detector based on an yttrium orthosilicate (YSO) scintillator coupled to a silicon photomultiplier (SiPM) array. When alpha particles interact with the YSO crystal, small flashes of light are produced and detected by the SiPM. The detector was tested using alpha particles from a ^238^Pu radioactive source over an energy range from approximately 0.4 MeV to 5.5 MeV.

The results showed that the detector responded in a stable and nearly linear way across the investigated energy range. Although its energy resolution was lower than that of conventional silicon detectors, the system demonstrated several practical advantages including compact size, mechanical robustness, operation under normal atmospheric conditions, and simpler electronics integration.

These characteristics suggest that YSO–SiPM detectors may be useful for portable radiation-monitoring systems, nuclear safeguards, contamination measurements, and other field-deployable nuclear-instrumentation applications where durability and practicality are important.

## 1. Introduction

Alpha-particle spectroscopy plays an important role in nuclear physics, environmental monitoring, nuclear safeguards, contamination assessment, and radiation-security applications. ^1– 3^ Accurate alpha-particle measurements are commonly required for isotope identification and characterization of radioactive materials. Silicon semiconductor detectors are widely considered the standard instrumentation for high-resolution alpha spectroscopy because alpha particles generate charge directly within the semiconductor material, resulting in excellent intrinsic energy resolution. ^4^ However, silicon detectors also exhibit several limitations including mechanical fragility, sensitivity to radiation damage, relatively high fabrication costs for large-area systems, and operational constraints associated with vacuum or carefully controlled environmental conditions. ^5, 6^

Scintillation detectors provide an alternative approach for alpha-particle measurements because of their mechanical robustness, scalability, fast timing properties, and compatibility with compact electronics. Conventional alpha-sensitive scintillators such as ZnS (Ag) and CsI (Tl) are frequently used because of their strong scintillation response to heavily ionizing particles. ^7, 8^ Nevertheless, these materials may suffer from disadvantages including long scintillation decay times, afterglow effects, hygroscopicity, and integration limitations in compact detector systems.

Recent developments in silicon photomultipliers (SiPMs) have enabled compact scintillation detector systems characterized by high photon-detection efficiency, low operating voltage, magnetic-field insensitivity, and fast timing capability. ^9– 13^ Compared with conventional photomultiplier tubes, SiPMs provide substantially improved compactness, mechanical stability, and simplified integration with modern digital electronics.

YSO scintillators possess several favorable properties for compact radiation-detection systems, including fast scintillation decay, non-hygroscopic behavior, mechanical stability, and emission spectra well matched to silicon photodetectors. ^14– 16^ In comparison with conventional silicon alpha detectors, scintillator–SiPM systems may offer improved operational robustness, higher tolerance to harsh environmental conditions, easier scalability to larger active areas, and simpler electronics integration. Furthermore, the relatively fast scintillation decay time of YSO supports high count-rate capability and timing applications important for practical radiation-monitoring instrumentation.

Despite these advantages, the alpha-particle response of YSO scintillators coupled to SiPM arrays has received comparatively limited investigation. The present study therefore evaluates the response characteristics of a YSO scintillator coupled to a multi-pixel photon counter (MPPC) array for alpha-particle spectroscopy in the approximate energy range from 0.4 MeV to 5.5 MeV. The primary objectives are to investigate detector linearity, achievable energy resolution, low-energy response, and applicability for compact alpha-spectrometric systems.

## 2. Methods

### 2.1 Detector configuration

A 10 × 10 × 10 mm ^3^ yttrium orthosilicate (YSO) scintillation crystal was optically coupled to a Hamamatsu S13360–3050 MPPC array using optical silicone grease. ^17^ The crystal dimensions were selected to match the active area of the photodetector array. White PTFE reflector tape was applied to all scintillator surfaces except the optical coupling surface in order to improve scintillation-light collection efficiency. The main properties of the YSO scintillator used in the experiment are summarized in Table 1.

**Figure 1.**
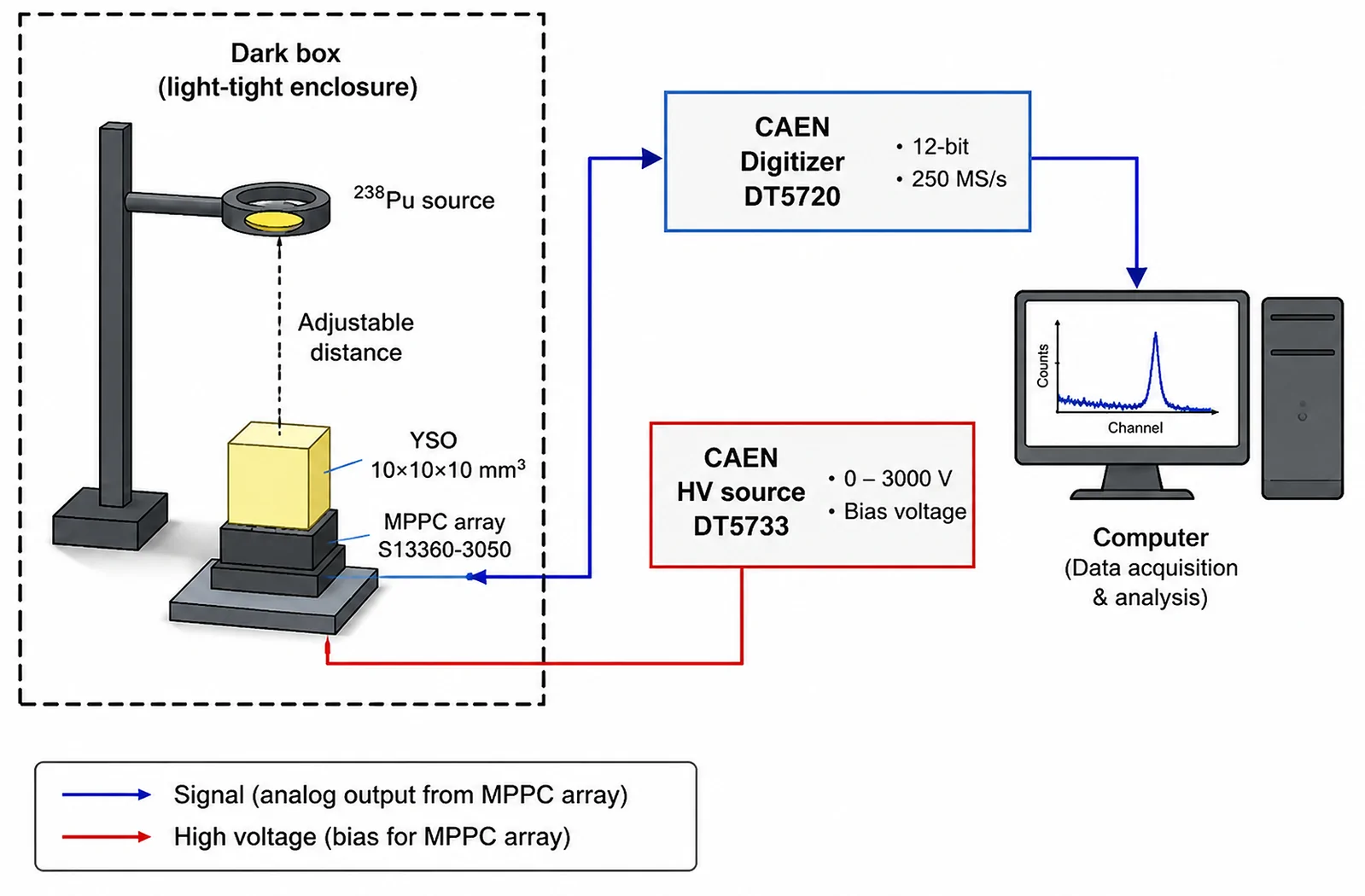
Experimental block diagram.

**Table 1.** Main parameters of the YSO scintillator.

Crystal	YSO
Density (g/cm ^3^)	4.4
Light Output (ph/MeV)	28,000
Emission Peak (nm)	410
Decay Constant (ns)	62
Refractive Index	1.82
Hygroscopicity	No
Anti-radiation (rad)	10 ^8^
Energy Resolution (% at 662 keV)	10–12
Hardness (Mohs)	5.8
Cleavage	No

The MPPC array consisted of sixteen SiPM elements operated from a common high-voltage bias channel. The detector was biased at 54.5 V, corresponding to the experimentally determined optimal operating voltage. Signals from all MPPC channels were combined into a single readout output.

### 2.2 Electronics and data acquisition

Figure 2 shows the experimental block diagram. Signal acquisition was performed using a CAEN DT5720 digitizer with 12-bit resolution and a sampling frequency of 250 MS/s. The bias voltage was supplied using a CAEN DT5733 high-voltage module. The detector assembly and radioactive source were positioned inside a light-tight and electromagnetically shielded enclosure.

**Figure 2.**
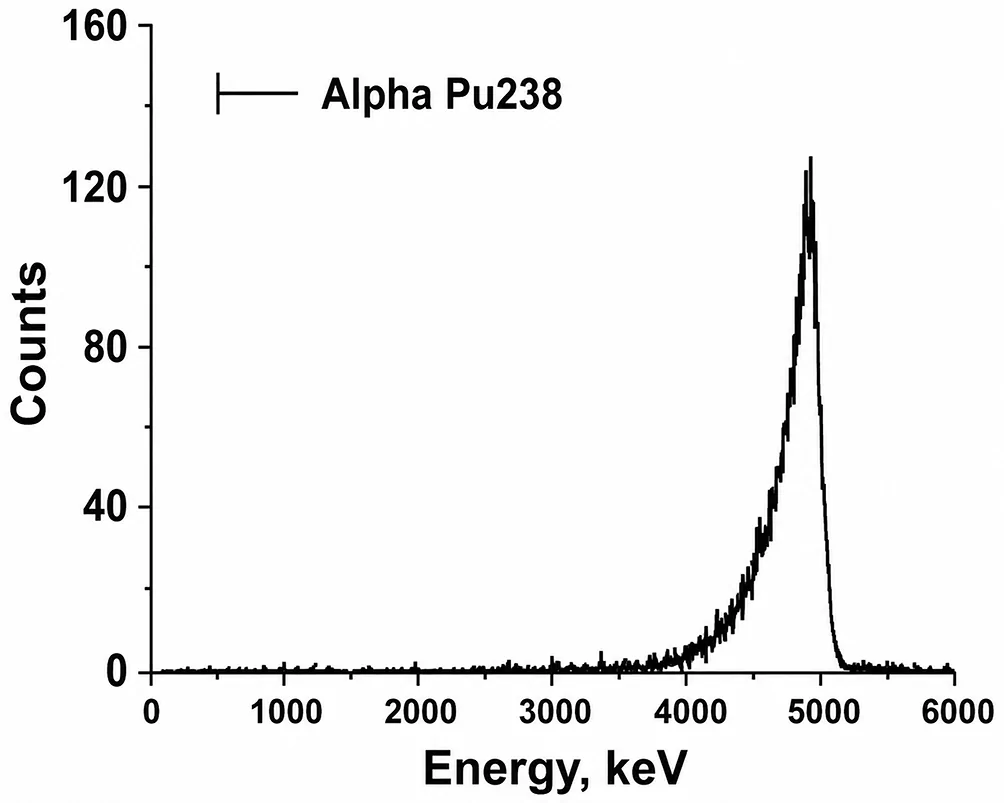
Measured energy spectrum of ^238^Pu at the initial position using Timepix detector.

Data acquisition was performed in self-trigger mode, while offline waveform processing and charge integration were carried out using ROOT-based software. ^18^ A charge-integration gate of 450 ns was selected according to the scintillation decay characteristics of the YSO crystal.

### 2.3 Alpha-particle measurements

An unsealed ^238^Pu alpha source was used for the measurements. The alpha-particle energy reaching the detector was controlled by varying the air-layer thickness between the source and detector. The source was mounted on an adjustable holder allowing controlled variation of the source-detector distance with an estimated positioning precision of approximately ±0.5 mm. Measurements were performed under ambient laboratory conditions at approximately 23 °C. The acquisition time for each measurement was fixed at 5 min. To validate alpha-particle propagation in air, SRIM simulations ^19^ were performed for 5500 keV alpha particles.

## 3. Results

As a reference measurement, the alpha-particle spectrum from the ^238^Pu source was initially measured using a Timepix hybrid pixel detector ^20– 22^ ( Figure 2, left). At the minimum source-detector spacing, the measured alpha peak energy was approximately 4915 keV, which is lower than the nominal alpha emission energy of approximately 5.5 MeV because of energy losses occurring before detection.

To estimate alpha-particle propagation and energy loss in air, SRIM simulations were performed for 5500 keV alpha particles. The calculated ionization-loss distribution and ion-range distribution are presented in Figure 3. The ionization profile shown in Figure 3 (left) demonstrates the progressive increase in energy loss near the end of the alpha-particle trajectory, corresponding to the Bragg peak region, where ionization density reaches its maximum immediately before the particles stop. The simulated ion-range distribution shown in Figure 3 (right) yielded a mean alpha-particle flight distance in air of approximately 40.6 mm with a longitudinal straggling of approximately 1.08 mm. The relatively narrow stopping distribution indicates small statistical fluctuations in alpha-particle propagation under the investigated conditions. These simulation results were used to estimate the expected alpha-particle energy degradation in air and to validate the experimental source-detector geometry.

**Figure 3.**
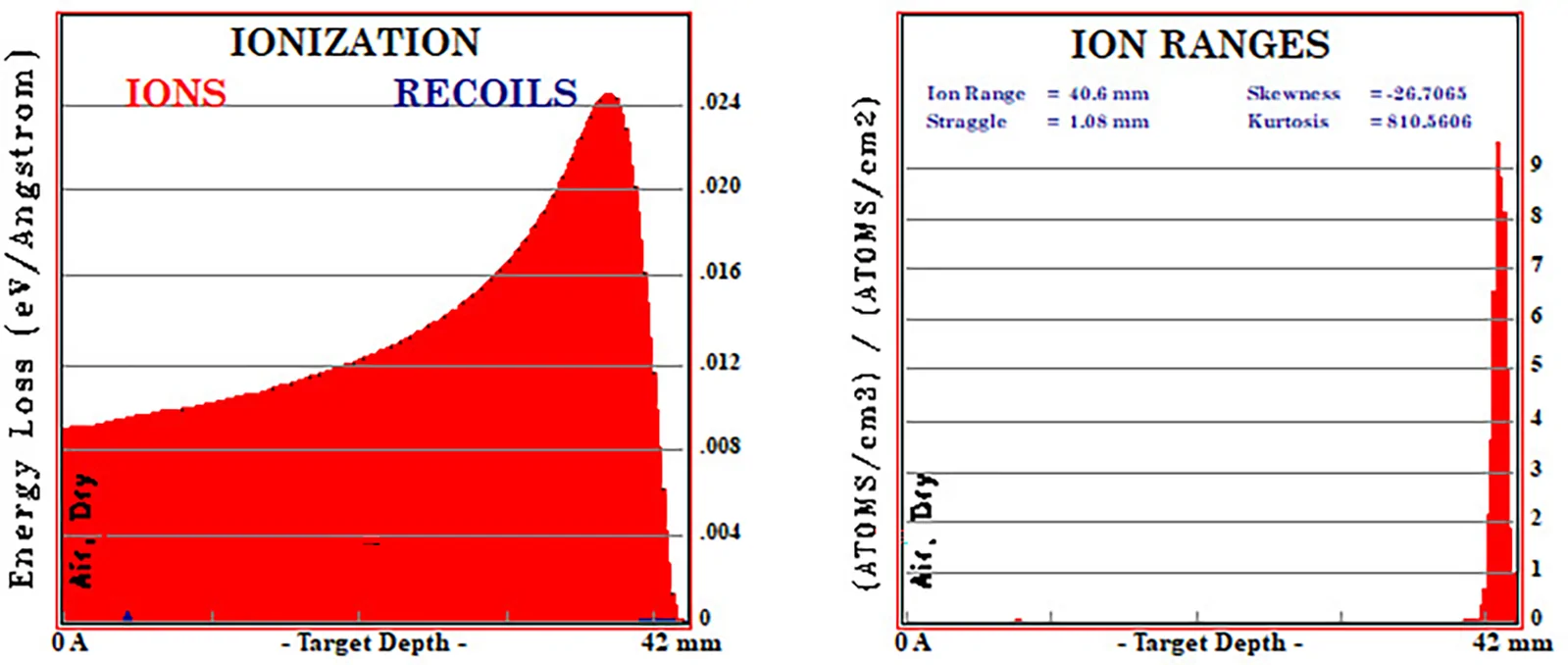
SRIM simulation results for 5500 keV alpha particles propagating in air: ionization energy-loss distribution exhibiting the Bragg peak behavior (left) and calculated ion-range distribution in air (right).

Measured alpha-particle spectra obtained at different source-detector distances are presented in Figure 4 (left). The measurement sequence was subsequently repeated using the YSO–SiPM detector system. Starting from a source-detector spacing of 2 mm, the air gap was increased in 5 mm increments, thereby progressively reducing the alpha-particle energy reaching the detector. Under the selected experimental conditions, alpha-particle peaks remained clearly distinguishable down to energies of approximately 500 keV. At energies below approximately 250 keV, alpha-induced signals increasingly overlapped with the gamma-induced background and could no longer be separated reliably. The detector response exhibited approximately linear behavior throughout the investigated energy interval, as shown in Figure 4 (right).

**Figure 4.**
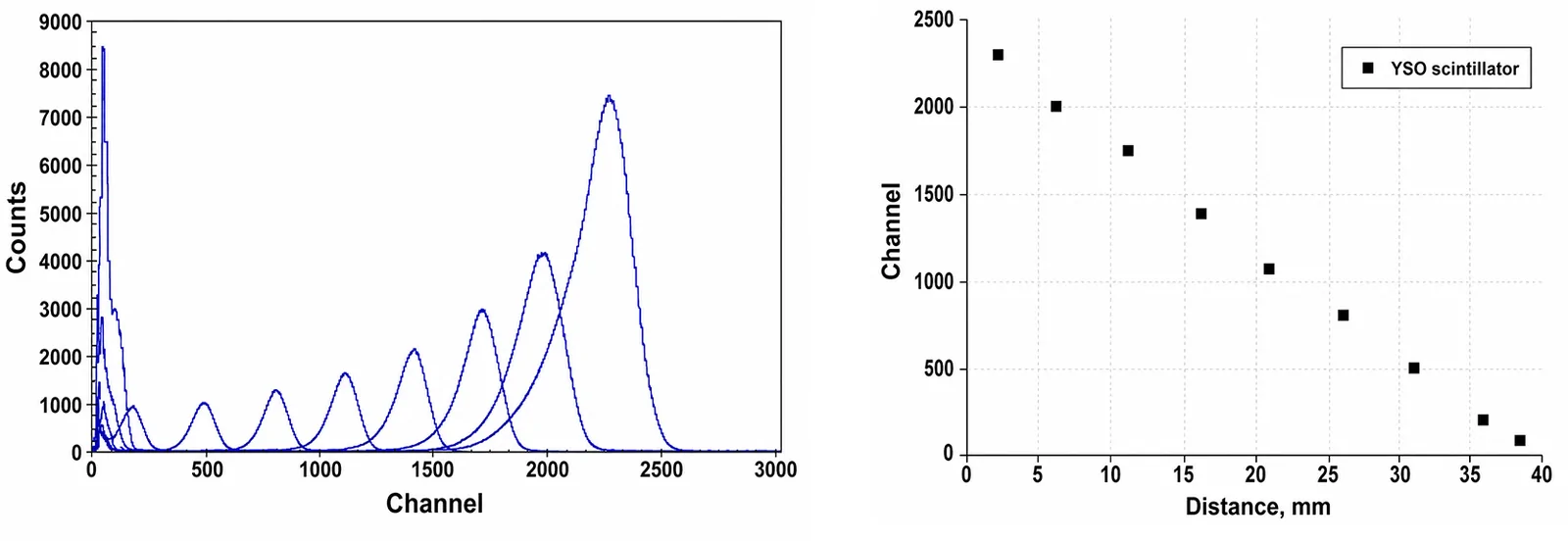
Measured alpha-particle spectra from ^238^Pu source with the YSO scintillator (left) and linearity of the channel-distance dependence (right).

The experimentally determined alpha-particle flight distance for 5500 keV alpha particles was measured as 40.58 ± 0.87 mm, which is consistent with the SRIM prediction. This level of agreement supports the consistency of experimental geometry and confirms that the YSO-MPPC configuration can track alpha-energy degradation in air with acceptable accuracy for practical spectrometric use.

The measured energy resolution varied from approximately 10.5% at 5.5 MeV to approximately 21% at alpha-particle energies near 0.4 MeV, as presented in Figure 5 (left). The estimated deviation from linearity across the investigated energy interval was approximately 125 keV, corresponding to a relative deviation of approximately 3%, as shown in Figure 5 (right).

**Figure 5.**
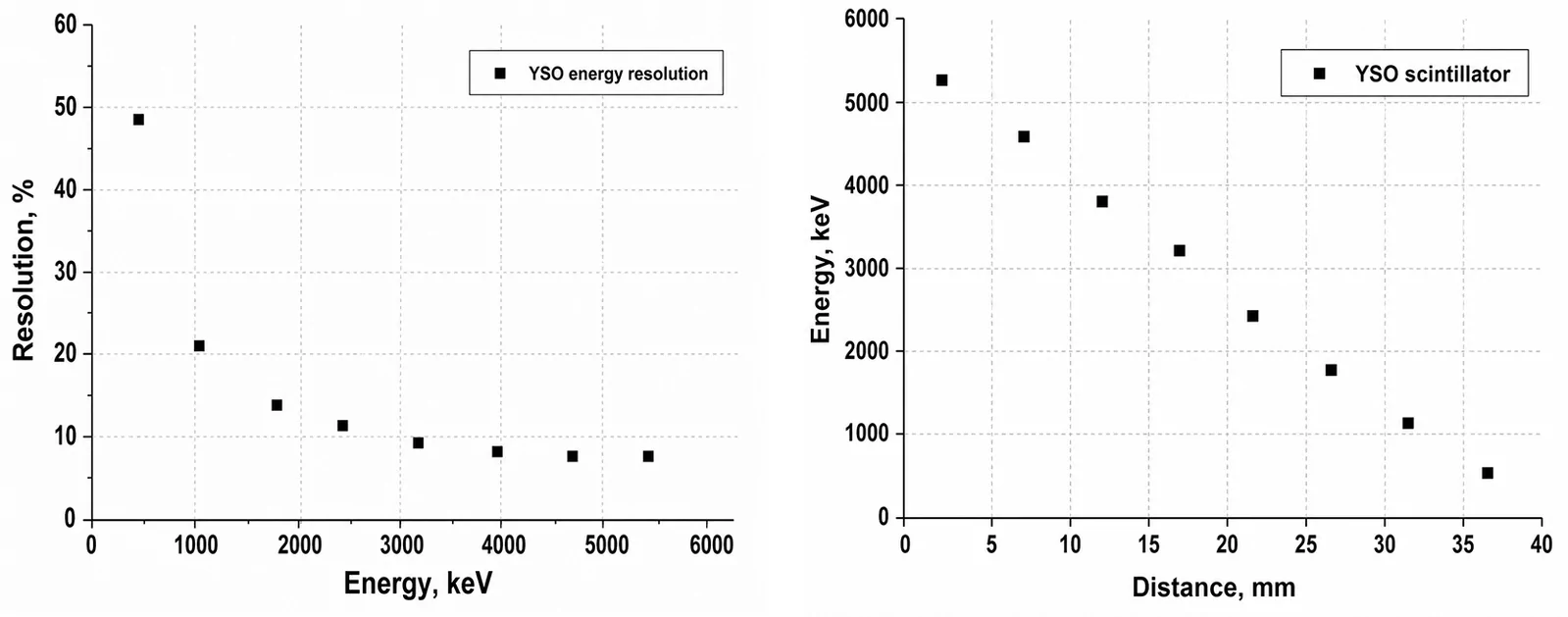
Dependence of energy resolution (left) and energy linearity (right) on alpha-particle energy from ^238^Pu.

The deterioration of energy resolution at lower energies is primarily attributed to reduced scintillation-photon statistics, increased relative contribution of electronic noise, and energy straggling in air. In addition, alpha-induced quenching effects in YSO scintillators may contribute to non-proportional scintillation response at low alpha-particle energies because of ionization-density-dependent quenching mechanisms.

## 4. Discussion

The obtained results demonstrate that the investigated YSO-SiPM detector configuration can provide practical alpha-particle spectrometric capability over a broad energy interval extending from approximately 0.4 MeV to 5.5 MeV. Compared with conventional silicon alpha-particle detectors, the investigated system provides substantially lower intrinsic energy resolution. Silicon detectors typically achieve superior spectrometric performance because alpha particles generate charge directly within the semiconductor material without intermediate scintillation processes. Nevertheless, scintillator-SiPM systems provide several practical advantages important for applied nuclear instrumentation. The investigated YSO-SiPM detector demonstrated stable operation under ambient conditions while providing improved mechanical robustness, simplified electronics integration, and scalability to larger active detection areas. In addition, the relatively fast scintillation decay time of YSO supports high count-rate capability and timing applications. These characteristics may be advantageous for portable radiation-monitoring systems, safeguards instrumentation, environmental contamination measurements, and other field-deployable nuclear-detection applications where practical operation and detector robustness are prioritized over ultimate spectrometric resolution.

The measured agreement between experimental alpha-particle flight distance and SRIM simulation further supports the reliability of the detector calibration and measurement geometry.

Several limitations remain. The present study did not investigate pulse-shape discrimination capability or detailed alpha-quenching behavior in the YSO scintillator. Furthermore, operation at lower alpha-particle energies was limited by increasing overlap between alpha-induced and gamma-induced signals.

Future investigations should therefore focus on optimization of scintillation-light collection, digital pulse processing, alpha/gamma discrimination capability, and detailed characterization of ionization-density-dependent quenching effects in YSO scintillators.

## 5. Conclusions

The response of a YSO scintillator coupled to a Hamamatsu MPPC array for alpha-particle spectroscopy was investigated in the approximate energy range from 0.4 MeV to 5.5 MeV. The detector exhibited stable and approximately linear response characteristics with an estimated deviation from linearity of approximately 3%. The measured energy resolution varied from approximately 10.5% at 5.5 MeV to approximately 21% at lower alpha-particle energies near 0.4 MeV. The experimentally measured alpha-particle flight distance in air was consistent with SRIM simulations.

Although the investigated detector does not provide the spectrometric resolution achievable with conventional silicon alpha detectors, the obtained results demonstrate that the YSO–SiPM configuration represents a compact and mechanically robust detector solution suitable for portable and field-deployable alpha-particle instrumentation.

## Ethics and consent

Ethical approval and consent were not required for this study.

## Data Availability

Open Science Framework: **Investigation of YSO–SiPM detector response for alpha spectroscopy applications.** DOI: https://doi.org/10.17605/OSF.IO/8W53H ^(29^ ^)^ This project contains the following underlying data:
• Alpha spectroscopy measurement data • Detector response datasets • Source data for all figures presented in the manuscript Data are available under the terms of the Creative Commons Attribution 4.0 International license (CC-BY 4.0).
