# Supplementary material for: Investigation of YSO–SiPM detector response for alpha spectroscopy applications
Source: Open Res Eur. 2026 Sep 18;6:357. [Version 1] doi: 10.12688/openreseurope.24095.1 (PMC13650315; doi:10.12688/openreseurope.24095.1)
Supplement: Peer review history

# Reviewer report: Reviewer response for version 1

**DOI:** 10.21956/openreseurope.26083.r81456

**Published:** 2026-10-16

**Authors:** Wataru Kada

**Affiliations:** 1 Faculty of Engineering, Tohoku University, Sendai, Miyagi, Japan

We would like to thank the Editor and the authors for giving us the chance to review the manuscript " **Investigation of YSO–SiPM detector response for alpha spectroscopy applications**,” on a relevant detector idea and shows some encouraging early results. The work has some useful aspects. These include the comparatively underexplored YSO–SiPM configuration, measurements over an apparent alpha-energy interval of about 0.4–5.5 MeV, and a comparison of the measured air range with SRIM calculations. A small, non-hygroscopic crystal and a SiPM-compatible readout may offer practical advantages. 

 However, the reported alpha-spectrometric performance and field applicability still need to be checked carefully. In particular, the energy assignment, calibration, linearity calculation, and resolution analysis are not defined sufficiently. I would suggest making some major changes.

 1. We need more information about the detector geometry and readout. The thickness of YSO (10 mm) is much more than the range of MeV alpha particles in YSO. Talk about the optical benefits of this, and whether it could make it more sensitive to gamma or X-rays. The reported 10 × 10 mm² crystal does not obviously match a 4 × 4 array of 3 × 3 mm² SiPM elements, whose active area is at least 12 × 12 mm² before gaps. 

 2. It would be ideal to have representative high- and low-energy waveforms, baseline information, and an integration-window. Show how it affects the collected charge, signal-to-noise ratio, and resolution.

 3. The energy resolution should be defined explicitly, ideally as FWHM/E × 100%, using the peak fitting method. The fit range, background model and uncertainty treatment should be stated for every energy. The text says 10.5% at 5.5 MeV and 21% near 0.4 MeV, but the plotted low-energy point looks much worse. The figure, data, energy labels and numerical values need to be checked. What's more, the observed peak width probably combines a few different effects:

 - alpha-energy straggling in air

 - source thickness

 - angular effects

 - source–detector positioning uncertainty

 - light-collection variation

 - SiPM statistics

 - electronic noise 

 These contributions should be measured or estimated. For now, the result should be identified as the overall resolution in the stated air-gap geometry, rather than the intrinsic YSO–SiPM resolution. The low-energy discussion should distinguish the lowest measured point near 0.4 MeV, the lowest clearly resolved alpha peak near 0.5 MeV, and the approximate 0.25 MeV region where separation becomes unreliable. Please measure the brightness of the gamma and background in this area.

 4. The application claims should match the measurements. The ability to tell the difference between different types of radioactive materials is limited by a problem that causes a difference in energy levels. This problem is about 10.5% at high energy levels but gets much worse at low energy levels. Please compare the results with silicon alpha detectors and other methods, including collimated silicon systems and scintillators such as ZnS (Ag) and CsI(Tl). 

 5. It seems that the measurements were done in a room that kept the light out, so we need to check how well these measurements work when there is more or less light outside. Unless it has been directly tested, the ability to withstand high levels of radiation, the performance of the timing system, the capability of the high-count rate and the ability to operate in difficult conditions should be described as potential advantages. 

 6. The entries in Table 1, for example i.e. "Anti-radiation (rad) 10^8" needs references, test conditions. Claims that silicon detectors are not suitable for use on the move should be backed up.

 7. The way that the energy of an incident is measured at each distance between the source and the detector must be explained. The study uses one Pu-238 source and changes the air gap, but it is unclear whether the assigned energies come from SRIM, Timepix measurements, a fitted range–energy relation, or a combination of these. The authors should explain the air composition, pressure, temperature, source-window effects, and any assumptions about dead layers. They should also explain the Timepix calibration and geometry, the minimum measurement distance, and the uncertainty and origin of the measured 4.915 MeV value relative to the nominal Pu-238 alpha energy near 5.5 MeV. The source dimensions, active deposit diameter, encapsulation, and collimation should be reported because they affect how much the beam spreads and how much its peak broadens.

 8. The claims about energy response and linearity need a calibration chain that can be traced. You can't use a plot of pulse channel against source-detector distance to establish energy linearity because alpha energy loss in air is nonlinear with distance. Please plot pulse channel against incident energy, state the calibration function, provide residuals, and define exactly how the reported 3% nonlinearity or 125 keV deviation was obtained. Figure 4 should clearly show that the data is from the channel-distance plot, not the energy-response or calibration plot. This should be obvious from the labels and caption. The numbers in the experimental block diagram and the Timepix spectrum should also be corrected, and Figure 3 would be better with extra lines and numbers on the axes.

 9. The experimental alpha range also needs a derivation that can be reproduced. Please explain how the measurement of 40.58 ± 0.87 mm was obtained. Was it calculated using endpoint extrapolation, peak disappearance, or a fit to channel versus distance? Explain how the fitting was done and how the uncertainty was calculated, especially given that the positioning was reported to be within ±0.5 mm. Make sense of the different values of 42 mm, 40.6 mm and 40.58 mm that appear in different parts of the manuscript.

 The updated version of the document would be much better if it included a measurement chain that could be repeated, figures and results that were all in line with each other, and an assessment of how likely it is that we can identify radionuclides that are present. 

 We hope these comments help the authors improve the work.

Is the study design appropriate and does the work have academic merit?

No

Is the work clearly and accurately presented and does it cite the current literature?

Partly

If applicable, is the statistical analysis and its interpretation appropriate?

Partly

Are all the source data underlying the results available to ensure full reproducibility?

Yes

Are the conclusions drawn adequately supported by the results?

Partly

Are sufficient details of methods and analysis provided to allow replication by others?

No

Reviewer Expertise:

Radiation measurement and applications

I confirm that I have read this submission and believe that I have an appropriate level of expertise to state that I do not consider it to be of an acceptable scientific standard, for reasons outlined above.

# Reviewer report: Reviewer response for version 1

**DOI:** 10.21956/openreseurope.26083.r81460

**Published:** 2026-10-10

**Authors:** Julien Venara

**Affiliations:** 1 CEA-Marcoule, Bagnols-sur-Cèze, France

This study investigates the potential of a YSO scintillator coupled to a SiPM array for alpha-particle spectroscopy at ambient air pressure. The work is of interest as it explores an alternative detection technology and provides experimental results on the detector response over a relatively broad range of alpha-particle energies. The results contribute to assessing the potential of this configuration for alpha detection and spectrometry applications. However, several points would benefit from clarification, particularly regarding the detector configuration, experimental methodology, and interpretation of the measured performances in relation to the intended applications. In particular, the consistency of the reported energy resolution, the determination of the alpha-particle range, and the discussion of the detector's actual spectrometric capabilities should be addressed to support the scientific conclusions.

**Methods**

 2.1 Detector configuration

 1. The authors indicate that the dimensions of the YSO crystal were selected to match the active area of the photodetector. While this choice is understandable in terms of light collection efficiency, could the authors clarify the choice of a 10 mm crystal thickness? Indeed, the range of alpha particles in YSO is expected to be well below 1 mm. Such a thickness may influence light collection and increase the detector sensitivity to gamma-ray background.

 2. The authors indicate that the YSO crystal was wrapped with PTFE reflector tape, except for the surface coupled to the photodetector. Could the authors clarify whether the PTFE tape was also applied to the alpha-particle entrance surface?

 3. The authors state that the 10 × 10 × 10 mm³ YSO crystal was matched to the active area of the photodetector, described as a 4 × 4 array of Hamamatsu S13360-3050 SiPMs. However, each SiPM has an active area of 3 × 3 mm², resulting in a minimum array size of 12 × 12 mm², excluding gaps between elements. Could the authors clarify the exact MPPC array configuration, its overall dimensions, and how the 10 × 10 mm² crystal was coupled to the photodetector?

 4. The authors state that the detector was biased at 54.5 V, corresponding to the experimentally determined optimal operating voltage. Could the authors clarify how this optimal voltage was determined, including the optimization criterion and the experimental conditions?

 2.2 Electronics and data acquisition

 5. The authors state that the charge integration window was set to 450 ns based on the YSO scintillation decay time, reported as 62 ns in Table 1. Could the authors clarify how this integration time was selected and whether its influence on the signal-to-noise ratio and energy resolution was evaluated?

 2.3 Alpha particle measurements

 6. Could the authors specify the dimensions of the alpha source, particularly the diameter of the radioactive deposit? This parameter may influence the angular distribution of alpha particles reaching the detector and, consequently, the energy resolution of the measured spectra.

**Results**

 7. Could the authors specify the minimum source-to-detector distance used to acquire the alpha-particle energy spectra with the Timepix detector?

 8. The authors report an experimentally determined alpha-particle range of 40.58 ± 0.87 mm for 5.5 MeV alpha particles. Could the authors clarify how this value was determined, particularly whether it was obtained by extrapolating the measured peak channel as a function of the source-to-detector distance? The method used to estimate the associated uncertainty should also be specified, especially considering the reported source-positioning uncertainty of ±0.5 mm.

 9. The energy resolution values reported in the text do not appear to be consistent with those presented in Figure 5 (left). Based on the figure, the energy resolution seems to be approximately 8% at 5.5 MeV and 78% at 0.4 MeV, while a resolution of 21% appears to correspond to an energy of approximately 1 MeV. Could the authors verify these values and correct any discrepancies between the text and the figure?

**Discussion and conclusions**

 10. Since silicon detectors are frequently mentioned as a reference technology for alpha spectroscopy, it would be useful to provide a quantitative comparison of the energy resolutions achieved with both detector technologies, taking into account the experimental conditions. The authors should also discuss how the energy resolution of the proposed YSO–MPPC detector may limit its ability to discriminate between alpha-emitting radionuclides with closely spaced emission energies and, consequently, its suitability for practical alpha spectroscopy applications.

 11. The authors highlight the potential use of the proposed detector in portable and field-deployable applications. However, the measurements were performed inside a light-tight enclosure. Could the authors clarify how the detector could operate under ambient light conditions, particularly regarding the protection of the alpha-particle entrance surface? The potential impact of such protection on energy loss and energy resolution should also be discussed, especially considering the relatively limited spectrometric performance and its implications for alpha-emitting radionuclide identification.

 12. The discussion could benefit from a broader comparison with previous developments in in situ alpha spectrometry at ambient air pressure, particularly studies using silicon detectors and collimation systems. Such approaches have already demonstrated the feasibility of alpha-emitting radionuclide identification under representative measurement conditions. Discussing these previous works would help position the proposed YSO–MPPC detector in relation to existing solutions, particularly regarding energy resolution, radionuclide discrimination capabilities, and potential field applications.

 I used ChatGPT (OpenAI, GPT-6) solely to assist with English translation and language improvement. The scientific assessment and comments are entirely my own.

Is the study design appropriate and does the work have academic merit?

Yes

Is the work clearly and accurately presented and does it cite the current literature?

Partly

If applicable, is the statistical analysis and its interpretation appropriate?

Partly

Are all the source data underlying the results available to ensure full reproducibility?

Partly

Are the conclusions drawn adequately supported by the results?

Partly

Are sufficient details of methods and analysis provided to allow replication by others?

Partly

Reviewer Expertise:

I am a senior research engineer at the French Alternative Energies and Atomic Energy Commission (CEA), specializing in non-destructive radiological characterization for nuclear applications. My expertise covers alpha, beta and gamma spectrometry, gamma imaging, detector design and response modelling using Monte Carlo simulations, and measurement data analysis. My work spans the development of innovative measurement methods, from initial design to experimental qualification under laboratory and operational conditions, particularly for nuclear decommissioning applications.

I confirm that I have read this submission and believe that I have an appropriate level of expertise to confirm that it is of an acceptable scientific standard, however I have significant reservations, as outlined above.

# Reviewer report: Reviewer response for version 1

**DOI:** 10.21956/openreseurope.26083.r81458

**Published:** 2026-10-08

**Authors:** Michael Holik

**Affiliations:** 1 Czech Technical University in Prague - Institute of Experimental and Applied Physics, Prague, Czech Republic; 2 Faculty of Electrical Engineering, University of West Bohemia in Pilsen (Ringgold ID: 48232), Pilsen, Plzeň Region, Czech Republic

**Reviewer Report**

 The manuscript presents a potentially useful and practically relevant investigation of YSO coupled to a SiPM array for alpha-particle detection. The experimental results indicate that the proposed detector can provide energy-dependent alpha-particle response over a broad range while offering the practical advantages of a compact scintillator and SiPM based configuration.

 The topic is relevant, particularly for applications where mechanical robustness, compactness and operation under ambient conditions may be more important than the high energy resolution achievable with conventional silicon alpha detectors. The experimental concept is straightforward and potentially useful. However, the current manuscript requires substantial clarification of the energy calibration procedure, the definition and interpretation of the reported linearity and energy resolution. 

**Decision: Major revision required**

 The following list of comments are intended to improve interpretation  of the work and quality of the reviewed paper (MAx - Major comments, MIx - Minor comments):

 —--------------------------------------------------------------------------------------------

**MA1 - The choice YSO scintillator geometry**

 The choice of the 10 × 10 × 10 mm³ YSO geometry should be discussed in greater detail. While the 10 × 10 mm² lateral dimensions appear to be motivated by matching the MPPC array, the 10 mm scintillator thickness is substantially larger than the range of several-MeV alpha particles in YSO. The increased thickness may improve scintillation-light collection and homogenize the light distribution over the MPPC array, but it also increases the detector volume and consequently its sensitivity to X-ray and gamma-ray background. This may be particularly relevant to the reported deterioration of alpha/gamma separation at low alpha-particle energies. The authors should discuss the trade-off between alpha detection efficiency, optical light collection/uniformity, photon-background sensitivity, and if possible, justify the selected thickness.

**MA2 - Charge-integration window**

 The choice of the 450 ns charge-integration window should be justified in greater detail. The authors are encouraged to provide representative digitized waveforms (documenting a typical detector response to 5 MeV and 0.5 MeV alpha particles) of the YSO–SiPM signals recorded with the CAEN DT5720. Such waveforms would help the reader assess the actual scintillation pulse shape, rise and decay characteristics, baseline fluctuation and signal-to-noise ratio. This would also provide experimental support for the selected 450 ns charge-integration window, which corresponds to approximately 7.3 times the reported 62 ns YSO decay constant.

**MA3 - Clarification of the reported energy resolution**

 The manuscript reports an energy resolution of approximately 10.5% at 5.5 MeV, increasing to approximately 21% at lower alpha-particle energies. It would be important to clarify whether these values represent the intrinsic energy resolution of the YSO–SiPM detector or the overall experimental resolution obtained under the particular measurement conditions. In the present experimental approach, the alpha-particle energy incident on the scintillator is determined indirectly from the source-to-detector distance and the corresponding energy loss in air. Consequently, the measured values may include not only contributions from scintillation-photon statistics, SiPM response and electronic noise, but also the energy straggling of alpha particles in air and the uncertainty associated with the source-detector geometry and positioning (which may become increasingly important at lower alpha-particle energies).

**MA4 - Reference Timepix measurement**

 More details should be provided on the Timepix reference measurement used to determine the alpha-particle energy, including the detector configuration, calibration procedure, measurement geometry and the uncertainty/accuracy of the obtained energy value. In particular, the authors should clarify how the reported 4915 keV was determined.

**MA5 - Clarification of the energy calibration and linearity analysis**

 The manuscript presents the dependence of the measured peak channel on the source-to-detector distance in Figure 4 and describes the detector response as approximately linear. However, the channel–distance dependence is not equivalent to an energy–channel calibration, since the alpha-particle energy loss in air is nonlinear with propagation distance. The authors should clarify how the alpha-particle energy corresponding to each source-to-detector distance was determined and how the reported approximately 3% deviation from linearity was calculated. The procedure used to convert the measured peak positions into an energy scale should be described in sufficient detail to assess the validity of the reported energy linearity (it is not clear if it was determined based on measured signal versus alpha-particle energy or simply as an agreement between the distance-dependent alpha energy and the assumed/SRIM energy-loss model).

**MA6 - Inconsistency of the reported energy resolution**

 There appears to be a discrepancy between the energy-resolution values stated in the text and those shown in Figure 5. The manuscript reports an energy resolution of approximately 21% at alpha-particle energies near 0.4 MeV, whereas the lowest-energy data point in Figure 5 appears to correspond to a substantially larger resolution. The authors should verify the plotted data and clarify the corresponding alpha-particle energy and FWHM value. 

**MI1 - Introduction - More suitable wording of YSO based solution benefits**

 The statements concerning the limitations of silicon alpha detectors in portable and harsh-environment instrumentation should be somewhat more carefully qualified, since compact and ruggedized silicon alpha spectrometers are also commercially available.

**MI2 - Introduction - Detailed overview on available alpha particle detection scintillator materials**

 The Introduction would benefit from a more quantitative comparison of YSO with other scintillators used for alpha-particle detection and spectroscopy. In particular, the authors should clarify what specific advantage of YSO motivates its selection over established materials such as ZnS(Ag), CsI and other modern scintillators coupled to SiPMs.

**MI3 - Figure 3 – axis labeling**

 The x-axes in Figure 3 are labeled only at the endpoints of the displayed range, which makes it difficult to quantitatively read the position and evolution of the simulated quantities. Additional tick marks and numerical labels (e.g. at regular 5 or 10 mm intervals) should be added to improve the readability and quantitative interpretation of the figure.

**MI4 - More information on peak fitting**

 The manuscript would benefit from a brief description of how the alpha-particle peak positions and FWHM values were obtained from the measured spectra. It would be useful to specify whether Gaussian or another fitting function was used, how the background was treated. This information is particularly important for the low-energy spectra, where the alpha peak increasingly overlaps with the background.

**MI5 - Count-rate and timing capabilities**

 The authors may consider slightly qualifying the statements regarding high count-rate capability and timing applications, since these aspects were not directly evaluated in the present study. For example, the fast scintillation decay of YSO could be described as a property favorable for or potentially beneficial to high-rate and timing applications.

Is the study design appropriate and does the work have academic merit?

Yes

Is the work clearly and accurately presented and does it cite the current literature?

Partly

If applicable, is the statistical analysis and its interpretation appropriate?

Partly

Are all the source data underlying the results available to ensure full reproducibility?

Partly

Are the conclusions drawn adequately supported by the results?

Partly

Are sufficient details of methods and analysis provided to allow replication by others?

Partly

Reviewer Expertise:

Design and development of nuclear instrumentation; Application of radiation detectors

I confirm that I have read this submission and believe that I have an appropriate level of expertise to confirm that it is of an acceptable scientific standard, however I have significant reservations, as outlined above.

# Reviewer report: Reviewer response for version 1

**DOI:** 10.21956/openreseurope.26083.r81452

**Published:** 2026-10-08

**Authors:** Shirkhan Humbatov

**Affiliations:** 1 Baku State University, Baku, Baku, Azerbaijan

**Review report for the article**

**Investigation of YSO–SiPM detector response for alpha spectroscopy applications**

 The manuscript presents an experimental investigation of a YSO scintillator coupled to a SiPM array for alpha-particle spectroscopy. YSO scintillators are more commonly employed in photon-detection applications, particularly gamma-ray spectroscopy and related radiation-detection systems, whereas their response and applicability for charged-particle detection have been investigated to a much more limited extent. From this perspective, the present study is relevant not only to the development of compact and robust radiation-detection systems, but also to extending the potential application of YSO-based scintillation detectors to charged-particle spectroscopy.

 The experimental results demonstrate an approximately linear detector response over a broad alpha-particle energy range and show reasonable agreement between the experimentally determined alpha-particle range in air and SRIM simulations. The manuscript is generally clear and the experimental approach is appropriate. However, several aspects should be clarified before indexing. In particular, the authors should provide a clearer description of the energy-calibration procedure, explain how the experimental alpha-particle range and its uncertainty were determined, define the method used to calculate the energy resolution, and resolve minor inconsistencies concerning the lowest detectable alpha-particle energy and the simulated alpha-particle range. Additional clarification of the MPPC readout configuration and improvement of several figure captions would further strengthen the manuscript.

 Minor presentation issues, including inconsistent terminology and figure numbering, should also be corrected. Overall, the manuscript presents useful experimental results and addresses a comparatively less explored application of YSO scintillators for charged-particle detection. The manuscript may be considered for indexing after the points listed below are adequately addressed.

  

1. The procedure used to assign alpha-particle energies to the different source-detector distances should be described more clearly. It should be stated whether the energies were determined from SRIM calculations, Timepix measurements, or another calibration procedure.
2. The reported SRIM range should be made consistent throughout the manuscript. The Abstract refers to approximately 42 mm, whereas the Results section reports approximately 40.6 mm.
3. The authors report an experimentally determined alpha-particle range of (40.58 \pm 0.87) mm. The method used to obtain this value and its uncertainty should be briefly explained.
4. The definition of energy resolution should be explicitly stated, for example whether it was calculated as FWHM/E. The peak-fitting procedure used to determine the resolution should also be briefly described.
5. The manuscript states that alpha-particle peaks remained distinguishable down to approximately 500 keV, while the investigated energy interval is reported as extending to approximately 0.4 MeV. The distinction between the lowest measured energy and the practical detection/identification threshold should be clarified.
6. Additional information about the electrical configuration of the 16 SiPM elements would improve reproducibility. The authors should briefly describe how the individual elements were combined into a single output and, if possible, provide the corresponding operating overvoltage.
7. The figure numbering should be checked. In Section 2.2, the experimental block diagram is referred to as Fig. 2, whereas the corresponding caption identifies it as Figure 1. In addition, the caption of Figure 4 refers to “channel-distance dependence,” although the discussion concerns detector energy response and linearity. The terminology and axis descriptions should be clarified.
8. Several parameters in Table 1 require clearer terminology or supporting references. In particular, the parameter “Anti-radiation (rad) 10⁸” should be clearly defined using standard terminology, and the reported energy resolution of 10–12% at 662 keV should include an appropriate reference or measurement conditions.

Is the study design appropriate and does the work have academic merit?

Yes

Is the work clearly and accurately presented and does it cite the current literature?

Yes

If applicable, is the statistical analysis and its interpretation appropriate?

Yes

Are all the source data underlying the results available to ensure full reproducibility?

Yes

Are the conclusions drawn adequately supported by the results?

Yes

Are sufficient details of methods and analysis provided to allow replication by others?

Yes

Reviewer Expertise:

My research expertise lies in physical electronics, optoelectronics, and the optical, dielectric, and electro-optical characterization of liquid crystals and nanoparticle composites. This background is relevant to assessing the optical and semiconductor-device aspects of the YSO–SiPM detector, particularly its characterization methods and experimental data interpretation.

I confirm that I have read this submission and believe that I have an appropriate level of expertise to confirm that it is of an acceptable scientific standard.

# Reviewer report: Reviewer response for version 1

**DOI:** 10.21956/openreseurope.26083.r81461

**Published:** 2026-09-30

**Authors:** Alaa Elbinawi

**Affiliations:** 1 Egyptian Atomic Energy Authority, Cairo, Egypt

The manuscript presents an experimental investigation of a YSO scintillator coupled to a SiPM/MPPC array for alpha-particle detection and spectroscopy. The topic is relevant to the development of compact radiation detectors, and the results show that the proposed YSO–SiPM configuration provides measurable and approximately linear responses over the investigated energy range. The comparison with SRIM calculations is also useful. Overall, the manuscript is generally well organized and clearly written.

 However, several methodological and interpretational points need clarification before the manuscript can be considered for indexing. In particular, the determination of alpha-particle energies, calibration procedure, energy-resolution analysis, background contribution, and the scope of the conclusions should be addressed.

1. **Alpha-energy determination:** Since a single ²³⁸Pu source was used and the alpha energy was varied by changing the air gap, the authors should explain more clearly how the alpha energy at each distance was determined and how the energy loss in air was calculated.
2. **Energy calibration and linearity:** The calibration procedure and the method used to obtain the reported 3% nonlinearity should be described more clearly. The authors should indicate the calibration method, fitting procedure, and how the reported deviation was calculated.
3. **Energy resolution:** Please define how the reported energy resolution was calculated and clarify whether it corresponds to FWHM/E. The procedure used to determine the peak width should also be stated.
4. **Timepix reference measurement:** The measured ²³⁸Pu energy of about 4.915 MeV is considerably lower than the nominal alpha energy. The authors should provide a clearer explanation of the energy losses responsible for this difference.
5. **Low-energy response:** The manuscript reports increasing overlap with the gamma background below approximately 250 keV. A brief quantitative discussion of the background and the practical low-energy detection limit would strengthen the results.
6. **Scope of the conclusions:** The reported energy resolution is poorer than that typically obtained with conventional silicon alpha detectors. The authors should clarify the specific practical advantages of the YSO–SiPM system and avoid overstating its suitability for alpha spectroscopy or harsh-environment applications in the absence of supporting measurements.
7. **High-count-rate capability:** The statement that YSO provides high-count-rate capability is reasonable based on its relatively fast decay time, but no count-rate or pile-up measurements are presented. This statement should therefore be appropriately qualified.
8. **Figure numbering:** Please check the figure numbering and descriptions, particularly Figure 2, which appears to be described differently in different sections of the manuscript.
9. **YSO radiation tolerance:** The term “Anti-radiation (rad)” in Table 1 should be clarified, properly defined, and supported by an appropriate reference.
10. **SRIM calculation:** Please specify the SRIM version used in the calculations.

Is the study design appropriate and does the work have academic merit?

Yes

Is the work clearly and accurately presented and does it cite the current literature?

Yes

If applicable, is the statistical analysis and its interpretation appropriate?

Partly

Are all the source data underlying the results available to ensure full reproducibility?

Partly

Are the conclusions drawn adequately supported by the results?

Yes

Are sufficient details of methods and analysis provided to allow replication by others?

Partly

Reviewer Expertise:

Nuclear Science, Radiation Detection & Scintillation Counting, Alpha & Gamma Spectrometry, Radiation Dosimetry, Radioprotection & Nuclear Safety, Reactor Physics.

I confirm that I have read this submission and believe that I have an appropriate level of expertise to confirm that it is of an acceptable scientific standard, however I have significant reservations, as outlined above.

# Reviewer report: Reviewer response for version 1

**DOI:** 10.21956/openreseurope.26083.r81454

**Published:** 2026-09-29

**Authors:** Ercan Yilmaz

**Affiliations:** 1 Bolu Abant Izzet Baysal University, Bolu, Turkey

To my best knowledge, the article is well written. The authors have reflected the applicability for alpha-parle article spectroscopy of the yttrium orthosilicate (YSO) scintillator. The article consists of scientific and technological novelty. The results demonstrate that the YSO-SiPM configuration represents a compact and mechanically robust detector solution for alpha-particle spectroscopy and its advantages. The methodology used is suitable. I advise the article for acceptance.

Is the study design appropriate and does the work have academic merit?

Yes

Is the work clearly and accurately presented and does it cite the current literature?

Yes

If applicable, is the statistical analysis and its interpretation appropriate?

Yes

Are all the source data underlying the results available to ensure full reproducibility?

Yes

Are the conclusions drawn adequately supported by the results?

Yes

Are sufficient details of methods and analysis provided to allow replication by others?

Yes

Reviewer Expertise:

My research area is about radiation sensors, semiconductor devices, scintillators

I confirm that I have read this submission and believe that I have an appropriate level of expertise to confirm that it is of an acceptable scientific standard.
